# Can teacher ostracism be prevented? Exploring how empowering leadership can mitigate teacher ostracism through work engagement

**DOI:** 10.1111/bjep.12745

**Published:** 2025-02-03

**Authors:** Alper Uslukaya

**Affiliations:** ^1^ Department of Educational Sciences, Faculty of Humanities and Social Sciences Çankırı Karatekin University Çankırı Turkey

**Keywords:** cross‐lagged effects, empowering leadership, teacher ostracism, work engagement

## Abstract

**Aim:**

Using the job demands‐resources model, this study theorizes the negative longitudinal relationship between empowering leadership and teacher ostracism, both directly and through work engagement.

**Method:**

For this purpose, data collected in three waves at four‐month intervals from 473 teachers (51.6% women; mean age = 42.26) working in schools at different levels in the centre of Elazığ province, eastern Turkey, during the 2022–2023 academic year were used. The relationships between the variables were analysed using a cross‐lagged panel model with latent variables.

**Results:**

The findings showed that empowering leadership positively related to work engagement and negatively related to teacher ostracism. Work engagement, in turn, is negatively related to teacher ostracism. Additionally, empowering leadership was found to be negatively related to teacher ostracism through work engagement.

**Conclusion:**

These results suggest that empowering leadership may be a crucial factor in preventing teacher ostracism, both directly and by enhancing employee engagement. The article concludes by discussing the theoretical contributions of the findings and presenting practical implications to help mitigate the risk of teacher ostracism.

## INTRODUCTION

Ostracism refers to the perception of an employee being isolated or rejected by other colleagues in the workplace (Ferris et al., 2008). It can manifest in various ways, such as avoiding eye contact, refusing social interaction, behaving silently, or ignoring greetings (Robinson et al., 2013; Zhu et al., 2017). Ostracism is a significant organizational phenomenon that is commonly experienced (Howard et al., 2020; Paşamehmetoğlu et al., 2022) and is more prevalent in high‐contact professions such as teaching (Williams et al., 2000). In Turkey, a country with a collectivist culture, the experiences of ostracism among teachers are particularly notable and have recently garnered increasing academic interest (Erdemli & Kurum, 2021; Polat et al., 2023). In collectivist societies, there is a high degree of in‐group belonging and control, and individuals who do not contribute to or behave in alignment with the group are punished with ostracism (Scott & Duffy, 2015). Additionally, due to the high level of mutual social dependency in such societies, employees are more sensitive to ostracism behaviours, leading to a deeper and more intense experience of ostracism (Sato et al., 2014).

Ostracism is less overt compared to explicit forms of mistreatment and is therefore often overlooked in organizations. This is concerning because ostracism undermines the needs for self‐esteem and belonging (Williams, 2007), which are crucial determinants of the quality of teachers' classroom practices and social relationships (Granjo et al., 2021). Moreover, O'Reilly et al. (2015) found in their fieldwork that organizations perceive ostracism as more acceptable and less harmful than harassment, but that ostracism actually has more devastating effects than harassment, underlining the risk it poses to employees and, therefore, teachers. Ostracism can also harm educational institutions and students. Research has shown that ostracism can disrupt social relationships within organizations (Huertas‐Valdivia et al., 2019) and threaten organizational performance by spreading counterproductive work behaviours (Yang & Treadway, 2018). Additionally, in a recent study, Uslukaya and Demirtaş (2020) found that teachers subjected to ostracism reflected the stress they experienced in the classroom, which in turn harmed classroom practices. The authors also found in the same study that these teachers struggled to establish healthy relationships with students and lost their ability to serve as positive role models.

Therefore, it is crucial to explore factors that can prevent teacher ostracism and develop effective interventions. However, research has generally focused on the dynamics that facilitate ostracism or its consequences (Paşamehmetoğlu et al., 2022), while the question of how ostracism can be prevented has been overlooked (Scott & Duffy, 2015). Preventing or managing ostracism is undoubtedly the responsibility of institutional school leaders, and there is some evidence that leadership behaviours can prevent ostracism. For example, in their study with hotel employees, Ali et al. (2020) found that leaders can reduce ostracism by fostering a supportive environment. Similarly, Babalola et al. (2017) revealed that leaders can prevent ostracism by cultivating a positive relational climate.

Moreover, some researchers have emphasized that individuals without personal power and those who perform poorly are potential victims of ostracism, indicating the importance of empowering leadership in preventing ostracism (Aquino & Lamertz, 2004; Howard et al., 2020; Khan et al., 2018). Empowering leadership is characterized by sharing power with followers, providing them with autonomy, and offering supportive behaviours (Cheong et al., 2019). Since this leadership style focuses on eliminating power constraints in the workplace to empower and engage employees and improve their performance (Wen et al., 2023), it can protect employees from negative workplace behaviours (Kim & Beehr, 2023). Despite this potential theoretical relationship between empowering leadership and ostracism, research has interestingly not examined this relationship.

**FIGURE 1:**
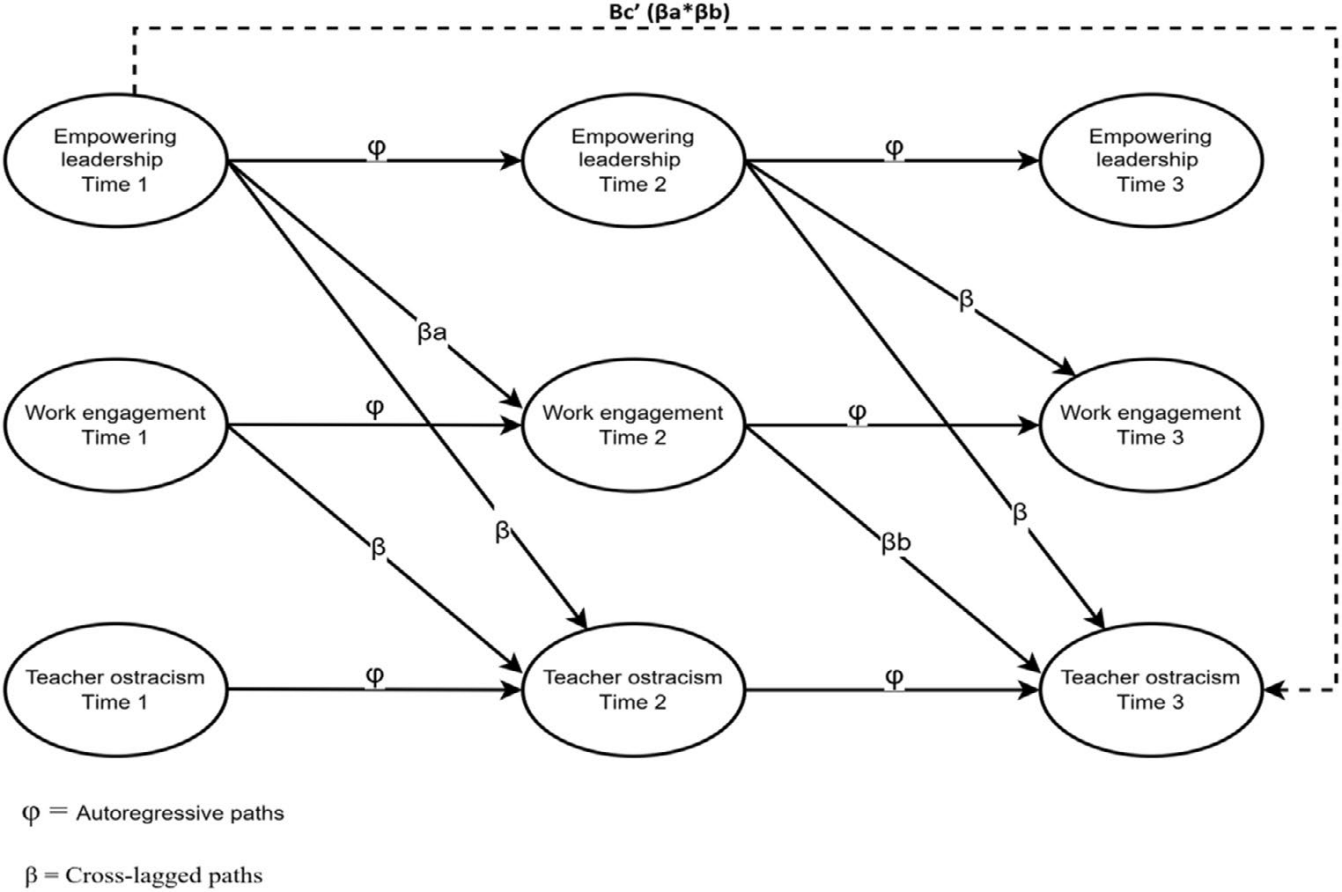
Theoretical model of the study.

This study employs the motivational pathway of the Job Demands‐Resources (JD‐R) model (Bakker & Demerouti, 2014) to explore the relationship between empowering leadership and ostracism. Based on the basic propositions of the model, it is suggested that empowering leadership is related to ostracism both directly and indirectly through work engagement. This relationship was analysed with a cross‐lagged panel model (CLPM) with latent variables using data collected from teachers in three waves. This study is expected to contribute to the literature in three ways. First, by establishing the empowering leadership‐ostracism relationship, it will expand the nomological network of these two constructs. Second, by operationalizing work engagement as a mediating mechanism between empowering leadership and ostracism, it will clarify the underlying mechanisms of ostracism. Finally, by demonstrating the relationships between key variables with time‐lagged evidence, it will provide robust evidence to the literature that has so far examined the leadership‐ostracism relationship through cross‐sectional studies (Ali et al., 2020; Babalola et al., 2017; Kanwal et al., 2019). Consequently, this study will offer important insights into efforts to prevent ostracism in educational settings, making significant contributions to the ostracism literature and providing policymakers and school principals with key strategies to prevent teacher ostracism. The model tested in this study is presented in Figure 1.

### Theoretical background and hypotheses

The JD‐R model is a groundbreaking theory in understanding occupational health and organizational behaviours (Bakker & Demerouti, 2017; Schaufeli & Taris, 2014). According to the model, two independent processes operate in the work environment: the strain process and the motivational process. The strain process is driven by job demands (e.g., workload, time pressure, emotional demands). High job demands, which are aspects of the job requiring constant effort, lead to the gradual depletion of employees' resources, resulting in negative well‐being and other adverse job outcomes (Bakker & Demerouti, 2014). In contrast, the motivational process is shaped by job resources (e.g., support, autonomy, leadership). Job resources, which are aspects of the job that facilitate goal achievement and promote personal growth and development, enhance employees' resource reserves, contributing to positive well‐being and favourable job outcomes (Bakker & Demerouti, 2014). In summary, the JD‐R model is an important conceptual framework linking work environment dynamics with employee well‐being and behaviour. Additionally, Howard et al. (2020) theorized in a comprehensive study that the characteristics of the work environment can either prevent or trigger ostracism. Therefore, integrating ostracism into the JD‐R model can help us better understand this phenomenon and identify connections that may help in preventing it.

The JD‐R model outlines a broad perspective on job resources, considering many positive job characteristics as job resources (Schaufeli & Taris, 2014). Empowering leadership, which focuses on employee empowerment (Cheong et al., 2019), has gained attention in the JD‐R literature and has called for research into its effects on employee well‐being and behaviour (Bakker & Demerouti, 2017). Empowering leadership is characterized by behaviours such as granting authority, sharing responsibilities, decision‐making, information sharing, skill development, and coaching for innovative performance (Konczak et al., 2000). This leadership style's focus on autonomy, coaching, and personal development has led some authors to consider it a primary antecedent of employee engagement (Tuckey et al., 2012; Wen et al., 2023). The JD‐R model posits that psychosocial resources like empowering leadership are key determinants of employee engagement (Bakker et al., 2023). According to the model, the autonomy, support, and performance feedback provided by the leader make employees feel valued and committed to the organization. If these positive feelings persist over time, they inspire and motivate employees, leading to increased engagement (Bakker & Demerouti, 2017). Consistent with this assumption, Alotaibi et al. (2020) found in their study with nurses that empowering leadership is a strong predictor of job engagement. Similarly, Wen et al. (2023) identified a positive relationship between empowering leadership and job engagement in their study with employees in the hotel industry.

According to the JD‐R model, when resources such as empowering leadership are available in work environments, positive behavioural outcomes are more likely; however, in the absence of resources, negative behaviours such as ostracism become more prevalent (Bakker & Demerouti, 2007). Insufficient resources, especially when job demands are high, trigger work‐related stress and helplessness, straining employees. Kim and Beehr (2023) found that the lack of adequate resources in high‐stress work environments leads to increased feelings of stress and helplessness, which negatively affect both employees' mental well‐being and their interactions with colleagues. Some studies have found that perpetrators try to mitigate the effects of these stressful situations by engaging in negative behaviours towards their colleagues, such as ostracism (Baillien et al., 2011). On the other hand, sufficient job resources can prevent many negative workplace behaviours, such as ostracism, by enhancing employees' energy and motivation and fostering a positive work environment (Bakker & Demerouti, 2007). The most striking feature of empowering leadership is that by providing autonomy and support, it improves employees' ability to control and influence their work environment and improves their performance (Cheong et al., 2019). This situation, which is characterized as empowerment, has been found to reduce ostracism in the workplace (Spreitzer, 1995). Indeed, perpetrators are particularly hesitant to ostracize empowered individuals (Howard et al., 2020), while they find it easier to ostracize non‐empowered individuals (Aquino & Lamertz, 2004). Based on these arguments, the following hypotheses are proposed:

Hypothesis 1 Empowering leadership positively related to work engagement.

Hypothesis 2 Empowering leadership negatively related to teacher ostracism.

Schaufeli et al. (2002) define work engagement, the central concept of the motivational pathway of the JD‐R model, as a positive and satisfying mental state related to work and conceptualize it through three constructs: vigour, dedication and absorption. Vigour is characterized by high levels of energy and resilience while working. Dedication refers to strong involvement in work, enthusiasm, significance, and a sense of challenge. Absorption denotes being fully concentrated, deeply immersed in work, and having difficulty detaching oneself from it (Bakker & Leiter, 2010). The JD‐R model acknowledges that work engagement corresponds to a dynamic process and that engaged employees are active creators. In other words, engaged employees are also motivated to remain engaged and thus are likely to foster supportive behaviours and build high‐quality relationships. Bakker and Demerouti (2018) suggest that this reciprocal nature of engagement—where engaged employees continually reinforce their energy and motivation—creates a self‐sustaining cycle of positive behaviour. As a result, engaged employees not only perform well but also contribute to a collaborative and supportive work environment. In this context, it can be expected that engaged employees will experience less ostracism in the workplace. Indeed, highly engaged employees stand out for their commitment, motivation, and performance. Such employees significantly contribute to teamwork (Bakker, 2022; Christian et al., 2011) and proactively enhance communication and collaboration, thereby strengthening their social ties at work (Schaufeli, 2015). This leads to recognition and social acceptance by other employees, reducing the likelihood of being ostracized (Bakker, 2008). Based on these theoretical explanations, the following hypothesis is proposed:

Hypothesis 3 Work engagement is negatively related to teacher ostracism.

The relationships summarized above indicate that there may be an indirect path between empowering leadership and ostracism through work engagement. The JD‐R literature, like leadership, assumes that job resources will shape work behaviours through work engagement as well as their direct relationship with job outcomes. For example, Schaufeli and Taris (2014) explain that work engagement plays a critical mediating role in the JD‐R model by linking job resources (such as leadership, autonomy, and support) with key workplace outcomes. Similarly, the literature on empowering leadership assumes that the impact of empowering leadership on workplace outcomes can be transmitted through various mediators (Cheong et al., 2019). These assumptions imply that empowering leadership can also prevent ostracism through work engagement. For example, teachers who are provided with autonomy and support by their leaders develop their motivation as they feel valued and appreciated. These feelings of motivation can increase their work engagement over time and thus reduce ostracism. This outcome is expected because engaged employees, who are appreciated and socially accepted due to their positive attitudes and productivity (Bakker, 2008), are less likely to experience ostracism compared to those who are not engaged. Moreover, there is empirical evidence that work engagement mediates the relationship between leadership and work outcomes. For example, Schmitt et al. (2016) found that work engagement mediates the relationship between transformational leadership and workplace behaviours. Based on this theoretical and empirical evidence, the fourth hypothesis is formulated as follows:

Hypothesis 4 Empowering leadership negatively related to teacher ostracism through work engagement.

## METHOD

In this study, the cross‐lagged panel model (CLPM) with latent variables was used to examine the time‐lagged relationships between key variables. CLPM is a widely used technique for examining time‐lagged relationships between variables with longitudinal data (Finkel, 1995). The time lags in CLPM, i.e., the time intervals between waves, are frequently discussed in the literature. While De Lange et al. (2004) suggest that one‐year intervals may be appropriate in work context models, Ployhart and Vandenberg (2010) emphasize that it would be a better choice to determine the time intervals in accordance with the nature of the variables rather than a fixed period. Some researchers, such as Taris and Kompier (2014), even argue that variables related to mood (e.g., work engagement) and perceptions (e.g., exclusion) can change rapidly in the short term, and therefore long time intervals (e.g., one year) are not necessary. Considering that the variables considered in this study may change in the short term due to the nature of the variables, it was deemed appropriate to set the interval between data collection waves as four months. This choice was made in accordance with the dynamic nature of the variables and aimed to capture temporal relationships accurately.

### Sample, procedure, and attrition analysis

The data used in this study were collected from teachers working in public schools located in the city centre of Elâzığ, a province in eastern Turkey, during the 2022–2023 academic year. A convenience sampling method was used to determine the sample. Convenience sampling is a non‐probability sampling method in which participants are selected based on specific criteria such as willingness, accessibility, and cost‐effectiveness (Etikan et al., 2016). However, significant efforts were made to diversify the sample as much as possible. For example, teachers from all educational levels (preschool, primary school, middle school, and high school) and different age groups were included in the study, and a gender balance was sought.

Initially, 100 easily accessible schools in this location were identified, and the administrators of these schools were contacted. After informing the school administrators about the purpose and scope of the research, 84 schools that granted permission for the implementation were listed. Surveys containing participants' demographic information and the scales used were prepared and placed in sealed envelopes. The first wave of data collection began in October 2022, and the designated schools were visited. After informing the teachers about the purpose and scope of the research, consent forms were signed by those who were willing to participate, and the surveys were administered. Within a three‐week period, 498 analysable data points were collected. Of the participants, 116 (23.2%) were employed in preschool, 125 (25.1%) in primary school, 138 (27.7%) in secondary school, and 119 (23.8%) in high school. In addition, 257 (51.5%) were women, 241 (48.3%) were men, and the mean age was 42.31 years (SD = 8.67). The second wave of data collection was conducted approximately four months after the first wave, in February 2023. Due to nine participants (1.8% dropout) voluntarily withdrawing from the study, 489 data points were collected in the second wave. The third wave of data collection took place four months after the second wave, in June 2023. During this wave, 16 teachers (3.2% dropout) voluntarily withdrew from the study, resulting in 473 data points collected.

To evaluate the potential bias introduced by teacher attrition, Little's MCAR test was conducted. The results showed that the missing data were completely at random (MCAR) concerning demographic variables (gender, age, school level) and the main study variables (empowering leadership, work engagement, teacher ostracism), *χ* ^2^(126) = 109.563, *p* = .851. Although the data were missing at random overall, a more detailed dropout analysis revealed that preschool teachers were significantly less likely to continue participating in the study after the second wave compared to teachers at other school levels, *χ* ^2^(3) = 14.22, *p* = .004. However, no systematic differences were found over time in terms of age or gender (*p*s > .05). Additionally, a series of *t*‐tests and ANOVA analyses were conducted to compare participants who remained in the study with those who dropped out across the three time points. The results indicated no significant differences in empowering leadership, work engagement, and teacher ostracism between groups at any time point. Effect sizes were negligible (*p*s > .05, *d*s < .01).

These findings collectively confirm that the exclusion of participants due to attrition did not introduce bias into the dataset. Therefore, the data from teachers who withdrew after Time 1 and Time 2 were excluded, and all analyses were conducted using the data from 473 teachers who participated in all three waves of data collection. Of the teachers whose data were analysed, 107 (22.6%) were in preschool, 121 (25.6%) were in primary school, 129 (27.3%) were in middle school, and 116 (24.5%) were in high school. Additionally, 244 (51.6%) were women, and 229 (48.4%) were men, with an average age of 42.46 (SD = 8.71). Considering the demographic characteristics of the participants, it can be said that the sample is consistent with the national teacher distribution in Turkey, according to the latest data from the Ministry of National Education (MoNE, 2021).

### Measures

#### Teacher ostracism

The Organizational Ostracism Scale, consisting of 14 items, including the isolation (*N* = 5) and exclusion (*N* = 9) dimensions, developed by Abaslı and Özdemir (2019), was used. An example item is: “During break times, they do not include me in their conversations.” Responses are given on a five‐point response scale ranging from 1 (never) to 5 (always), and high scores indicate high levels of ostracism. The Cronbach's Alpha values for both the isolation dimension (*α*_T1_ = .828, *α*_T2_ = .863, *α*_T3_ = .910) and the exclusion dimension (*α*_T1_ = .877, *α*_T2_ = .913, *α*_T3_ = .917) indicated a high level of internal consistency across all time points.

#### Work engagement

The nine‐item short version of the Utrecht Work Engagement Scale (UWES; Schaufeli et al., 2006) was used. The scale consists of three dimensions: vigour, dedication, and absorption, each containing three items. An example item is: “I get carried away with my work.” The responses are evaluated on a seven‐point rating scale ranging from 0 (never) to 6 (every day), and high scores indicate a high level of work engagement. The Cronbach Alpha values indicated a high level of internal consistency for the dimensions of vigour (*α*_T1_ = .748, *α*_T2_ = .857, *α*_T3_ = .844), dedication (*α*_T1_ = .720, *α*_T2_ = .861, *α*_T3_ = .836), and absorption (*α*_T1_ = .734, *α*_T2_ = .855, *α*_T3_ = .831) across all time points.

#### Empowering leadership

The 17‐item scale developed by Konczak et al. (2000) and adapted into Turkish by Konan and Çelik (2018) was used. The dimensions consist of granting authority (*N* = 3), responsibility (*N* = 3), and support (*N* = 11). A sample item on the scale is as follows: “My school principal frequently provides me with opportunities to develop new skills.” The responses are given on a five‐point response scale ranging from 1 (never) to 5 (always), and higher scores indicate a higher level of empowering leadership. The Cronbach's Alpha values indicated a high level of internal consistency for the dimensions of granting authority (*α*_T1_ = .769, *α*_T2_ = .773, *α*_T3_ = .824), responsibility (*α*_T1_ = .793, *α*_T2_ = .780, *α*_T3_ = .846), and support (*α*_T1_ = .900, *α*_T2_ = .899, *α*_T3_ = .928) at three different time points.

#### Covariates

The age, gender, and school level of the employees were controlled in this study because the literature clearly shows the relationship between these factors and ostracism (Erdemli & Kurum, 2021; Howard et al., 2020). For this purpose, dummy variables were created for gender (women = 0, men = 1), school level (preschool = 0, others = 1; primary school = 0, others = 1; secondary school = 0, others = 1; high school = 0, others = 1), and age (40 years and under = 0, over 40 years old = 1). While determining the age categories, Levinson's (1986) age curve was used as a basis. The author defines the age range of 20–40 as early adulthood, 40–65 as middle adulthood, and those over 65 as late adulthood. Since none of the participants in the study were over 65 years old, a dummy variable for late adulthood was not created.

### Statistical analyses

Before testing the hypotheses, several preliminary analyses were conducted using SPSS 27.0, including descriptive statistics, correlation analyses, and an examination for common method bias. Subsequently, Mplus 8.11 was used to assess the dataset's suitability for multilevel analysis, the adequacy of the sample, and to test for longitudinal invariance. In the final stage, after testing a general measurement model that included all latent variables using Mplus 8.11, structural equation modelling (SEM) analyses were performed to determine the cross‐lagged paths. To examine structural relationships, four different structural models were tested. First, a basic stability model that only controls for autoregressive paths was estimated (M1). Second, a model that includes both autoregressive paths and cross‐lagged paths from empowering leadership to teacher ostracism was tested (M2). Third, a model incorporating autoregressive paths, as well as cross‐lagged paths from empowering leadership to both teacher ostracism and work engagement, was tested (M3). Finally, a model including autoregressive paths, cross‐lagged paths from empowering leadership to work engagement and teacher ostracism, as well as cross‐lagged paths from work engagement to teacher ostracism, was examined (M4).

The Comparative Fit Index (CFI), Tucker‐Lewis Index (TLI), and Root Mean Square Error of Approximation (RMSEA) values were reported to examine the overall quality and fit of the hypothesized and alternative models. An RMSEA value less than .08 and CFI and TLI values of .90 and above signify acceptable model fit (Hu & Bentler, 1999). Additionally, following the recommendation by Chen et al. (2008), it is required that the lower limit of the 90% confidence interval for RMSEA be less than .05, and the upper limit be less than 1. In model comparisons, chi‐square difference tests were used, and changes in CFI and RMSEA were considered to assess longitudinal invariance. The size of the cross‐lagged effects was estimated by converting the regression coefficients into correlation metric using sample size and the Z‐values computed from the standardized coefficients and their associated standard errors (Rosenthal, 1994). This approach was chosen to allow for a uniform and easy scaling of the effects and to make them directly interpretable as a correlations (Peterson & Brown, 2005). Z‐transformed regression coefficients facilitate the comparison of effect sizes across different contexts or studies (Rosenthal, 1994). This is particularly useful in longitudinal research, where the magnitude of relationships may vary over time or across variables. Overall, these statistical procedures and fit indices ensure the reliability and validity of the data analyses and support the conclusions drawn from the study.

Mediation analysis was conducted using the MODEL CONSTRAINT command in Mplus, and bias‐corrected confidence intervals (CIs) obtained from bootstrapping procedures were controlled. It has been interpreted that CIs not including the zero value indicate that the estimates are statistically significant (Preacher & Hayes, 2008). While analysing all models, correlated errors of corresponding factors were linked. This is recommended to more accurately estimate the variance arising from the measurement situation and prevent measurement errors from inflating stability paths (Anderson & Williams, 1992). Similarly, the errors of indicators over time were also correlated. This procedure is necessary for models to exhibit better fit and for more accurate estimations (Newsom, 2004).

## RESULTS

### Descriptive statistics and correlations

Table 1 presents the descriptive statistics of the main variables and their correlation coefficients. A MANOVA analysis was conducted to evaluate systematic differences across the three waves. The results indicate that there was no significant difference in teacher ostracism, *F*_(2,1416)_ = 2.206, *p* > .05, while significant differences were observed in empowering leadership, *F*_(2,1416)_ = 4.389, *p* < .05 and work engagement, *F*_(2,1416)_ = 36.758, *p* < .001. Post‐hoc analyses revealed that empowering leadership in the third wave was significantly higher than in the first wave (*p* < .05), and work engagement in the second and third waves was significantly higher than in the first wave (*p* < .001).

**TABLE 1:** Descriptive statistics and correlation coefficients between variables.

Variables	Mean	SD	TO_T1_	WE_T1_	EL_T1_	TO_T2_	WE_T2_	EL_T2_	TO_T3_	WE_T3_	EL_T3_
TO_T1_	2.85 (2.84)	.74 (.74)	1								
WE_T1_	3.26 (3.27)	.72 (.73)	−.42** (−.41)	1							
EL_T1_	3.59 (3.58)	.72 (.73)	−.66** (−.66)	.58** (.57)	1					‐	
TO_T2_	2.74 (2.75)	.92 (.93)	.84** (.84)	−.54** (−.54)	−.76** (−.75)	1					
WE_T2_	3.76 (3.75)	1.07 (1.06)	−.47** (−.46)	.75** (.74)	.59** (.58)	−.60** (−.61)	1				
EL_T2_	3.66 (3.67)	.72 (.72)	−.56** (−.56)	.56** (.56)	.68** (.68)	−.69** (.69)	.61** (.62)	1			
TO_T3_	2.72 (2.72)	1.13 (1.12)	.77** (.76)	−.58** (−.59)	−.76** (−.75)	.90** (.89)	−.63** (−.64)	−.70** (−.69)	1		
WE_T3_	3.74 (3.74)	1.18 (1.18)	−.60** (−.61)	.84** (.83)	.71** (.70)	−.76** (−.77)	.80** (.80)	.70** (.70)	−.79** (−.79)	1	
EL_T3_	3.73 (3.72)	.85 (.86)	−.72** (−.71)	.61** (.61)	.86** (.87)	−.85** (−.84)	.64** (.65)	.78** (.79)	−.85** (−.85)	.81** (.81)	1
SD			.74 (.74)	.72 (.73)	.72 (.73)	.92 (.93)	1.07 (1.06)	.72 (.72)	1.13 (1.12)	1.18 (1.18)	.85 (.86)
Minimum			1.36	.67	1.18	1.07	.89	1.24	1.14	1.11	1.29
Maximum			4.93	5.44	4.65	4.64	5.89	4.88	5	6	4.94
Skewness			.51	−.83	−.97	.55	−.34	−.76	.67	−.51	−1.04
Kurtosis			−.63	1.01	.33	−1.07	−.45	.62	−1.09	−.64	.26
ICC			.02	.01	.03	.01	.01	.02	.01	.02	.03

*Note*: The coefficients in parentheses are the values of the simulation data set.

Abbreviations: EL, empowering leadership; ICC, intra‐class correlations; SD, standardized deviation; TO, teacher ostracism; WE, work engagement.

** *p* < .01.

The correlation coefficients show that, across all three time points, teacher ostracism is negatively related to both empowering leadership and work engagement, whereas work engagement is positively related to empowering leadership. These results indicate meaningful interrelationships between these constructs, emphasizing the potential necessity of additional research into their dynamic relationships across time. Furthermore, the fact that the correlation coefficients between independent variables were estimated to be below .80 suggests that there is no multicollinearity issue in the developed model (Bowen & Guo, 2011). Table 1 also presents intra‐class correlation coefficients (ICC). ICC values are obtained by dividing the variance at the group level by the total variance, and when the ICC values are estimated to be higher than .05, multilevel analyses are required (Hox et al., 2017). However, all ICC values for the variables were estimated to be lower than .05, indicating that the variance in these variables stems more from individual differences rather than differences between groups. Therefore, the analyses in the current study were conducted at the individual level.

### Tests of common method bias

The data used in the study were collected through self‐report scales. However, Podsakoff et al. (2012) argue that collecting data from the same participants may lead to common method bias, which could result in the estimated relationships between the measured variables appearing stronger or weaker than they actually are. Harman's (1967) one‐factor analysis was performed to assess the risk of common method bias. The results indicated that the one‐factor models explained less than 50% of the total variance at all three time points (T1 = 34.07%, T2 = 41.45%, and T3 = 44.76%). These findings suggest a low risk of common method bias and support the notion that the variance in the longitudinal data set is attributable to the variables (Podsakoff et al., 2012). Consequently, the path coefficients estimated between the variables are expected to be reliable and valid.

### Test of sample adequacy

Since the model of the current study is large and complex, and relatively large samples are needed to make robust predictions in such models (Muthén & Muthén, 2002), the adequacy of the sample of the current study was analysed. For this, a new dataset with 1000 samples equivalent to the original dataset was created using Monte Carlo simulation in Mplus, and the results were compared (Table 1). The results show that the parameter estimates and standard errors of the original dataset and the simulated dataset are estimated to be quite close to each other, and deviations do not exceed the 10% threshold. Therefore, it suggests that the original dataset is equivalent to a dataset with 1000 samples and has a relatively adequate size (Muthén & Muthén, 2002).

### Tests of longitudinal invariance

Longitudinal invariance tests were performed to determine whether the measurements captured the same structures across the three time waves. The analysis of longitudinal invariance started with a basic model (configural model) where no invariance constraints were applied. Then, models in which factor loadings (metric model), factor loadings and intercepts (scalar model), and factor loadings, intercepts, and unique factor variances (strict model) were constrained across time were tested, respectively (Widaman et al., 2010). Longitudinal invariance was evaluated based on the criteria recommended by Cheung and Rensvold (2002), which specify that a decrease in CFI of less than .01 and an increase in RMSEA of less than .015 indicate support for invariance. The results of the longitudinal invariance tests for empowering leadership, work engagement, and teacher ostracism are shown in Table 2.

**TABLE 2:** Longitudinal invariance indices of the variables used in the study.

	*χ* ^2^ (*df*)	CFI	TLI	RMSEA (90% CI)	Δ*χ* ^2^	Δ*df*	ΔCFI	ΔRMSEA
Teacher ostracism								
Configural model	1178.788 (762)	.981	.979	.034 (.032–.035)				
Metric model	1227.079 (786)	.980	.978	.034 (.033–.035)	48.291**	24	.001	.000
Scalar model	1265.838 (810)	.980	.978	.034 (.033–.035)	38.759*	24	.000	.000
Strict model	1356.121 (845)	.975	.973	.039 (.037–041)	90.283***	35	.005	.005
Work engagement								
Configural model	654.847 (261)	.964	.951	.056 (.049–.059)				
Metric model	687.437 (273)	.962	.951	.057 (.052–.061)	32.590**	12	.002	.001
Scalar model	744.195 (285)	.957	.948	.058 (.054–.062)	56.758***	12	.005	.003
Strict model	835.484 (303)	.949	.941	.059 (.055–.064)	91.289***	18	.008	.001
Empowering leadership								
Configural model	2789.049 (1137)	.947	.940	.055 (.050–.060)				
Metric model	2833.828 (1165)	.946	.941	.055 (.051–.060)	44.779*	28	.001	.000
Scalar model	2880.417 (1193)	.945	.942	.055 (.051–.060)	46.588*	28	.001	.000
Strict model	2983.574 (1233)	.941	.938	.058 (.057–.063)	103.157***	40	.004	.003

Abbreviations: CFI, comparative fit index; RMSEA, root mean square error of approximation; TLI, Tucker‐Lewis index.

* *p* < .05.

** *p* < .01.

*** *p* < .001.

The results indicate that the configural models created for all measurements demonstrate good fit indices. These findings suggest that the first condition of longitudinal invariance is met. All models with progressively increasing constraints also generally show acceptable fit indices. Additionally, the differences in CFI and RMSEA values (ΔCFI and ΔRMSEA) between each model and the preceding model being estimated are less than .010 for CFI and less than .015 for RMSEA, indicating that all measurements meet the conditions of longitudinal invariance (Chen, 2007). These results provide evidence that all measurements consistently and reliably capture the same constructs across the three data collection points, supporting the reliability of conducting longitudinal analyses.

### Cross‐lagged panel analysis

Table 3 shows the fit indices for the models constructed for the CLPM. Before testing the hypotheses, a general measurement model (Mmeasurement) is tested in which the indicators measured across the three time periods are related to the corresponding latent variables.

**TABLE 3:** The goodness‐of‐fit indices for the tested models.

Models	*χ* ^2^(*df*)	*p*	CFI	TLI	RMSEA (90% CI)	Δ*χ* ^2^	Δ*df*
M_(measurement)_	10,741.804 (6849)	.000	.939	.942	.035 (.033–.036)		
M1_(autoregressive)_	11,919.065 (6949)	.000	.925	.923	.039 (.038–.040)		
M2_(EL→TO)_	11,805.232 (6947)	.000	.927	.925	.038 (.037–.040)	113.833***	2
M3_(EL and WE→TO)_	11,763.128 (6947)	.000	.927	.925	.038 (.037–.039)	42.104***	2
M4_([EL and WE→TO] + [EL→WE])_	11,578.084 (6945)	.000	.930	.928	.038 (.036–.039)	185.044***	2
M4_([EL and WE→TO] + [EL→WE])_ ^a^	12,619.326 (7309)	.000	.920	.919	.039 (.038–.042)	1041.242***	364

Abbreviations: CFI, comparative fit index; EL, empowering leadership; RMSEA, root mean square error of approximation; TLI, Tucker‐Lewis index; TO, teacher ostracism; WE, work engagement.

^a^ Model with control variables (gender, age and school level).

*** *p* < .001.

The results indicate that the measurement model has generally acceptable fit values, *χ* ^2^(6849) = 10,741.804, TLI = .939, CFI = .942, RMSEA = .035 (90% CI [.033, .036]). This result shows that the latent variables in the longitudinal model have strong structural relationships with the observed variables in an accurate and consistent manner. Four different models were then tested to reveal the structural relationships between the latent variables. The model that provided the best fit to the data among the four structural models, as shown in Table 3, was Model 4, which was constructed based on the hypotheses *χ* ^2^(6945) = 11,578.084, CFI = .930, TLI = .928, RMSEA = .038 (90% CI [.036, .039]). The chi‐square difference test also confirmed that Model 4 is the best model (Δ*χ* ^2^ = 185.044, Δ*df* = 2, *p* < .001). Thus, the model (M4), in which empowering leadership is associated with work engagement and teacher ostracism, and work engagement is associated with teacher ostracism, received the strongest empirical support. In the last stage, the model (M4^a^) in which control variables (gender, age, and school level) were included in the M4 model was tested, and it was observed that this model also had acceptable fit values *χ* ^2^(7309) = 12,619.326, CFI = .920, TLI = .919, RMSEA = .039 (90% CI [.038, .042]). However, the chi‐square difference test showed that the fit of M4 worsened statistically when control variables were added (Δ*χ* ^2^ = 1041.242, Δ*df* = 364, *p* < .001). This may be related to the decrease in the parsimony of the model. However, the fact that the model still has acceptable fit values indicates that the general structure of the model can be preserved.

### Cross‐lagged relationships

**FIGURE 2:**
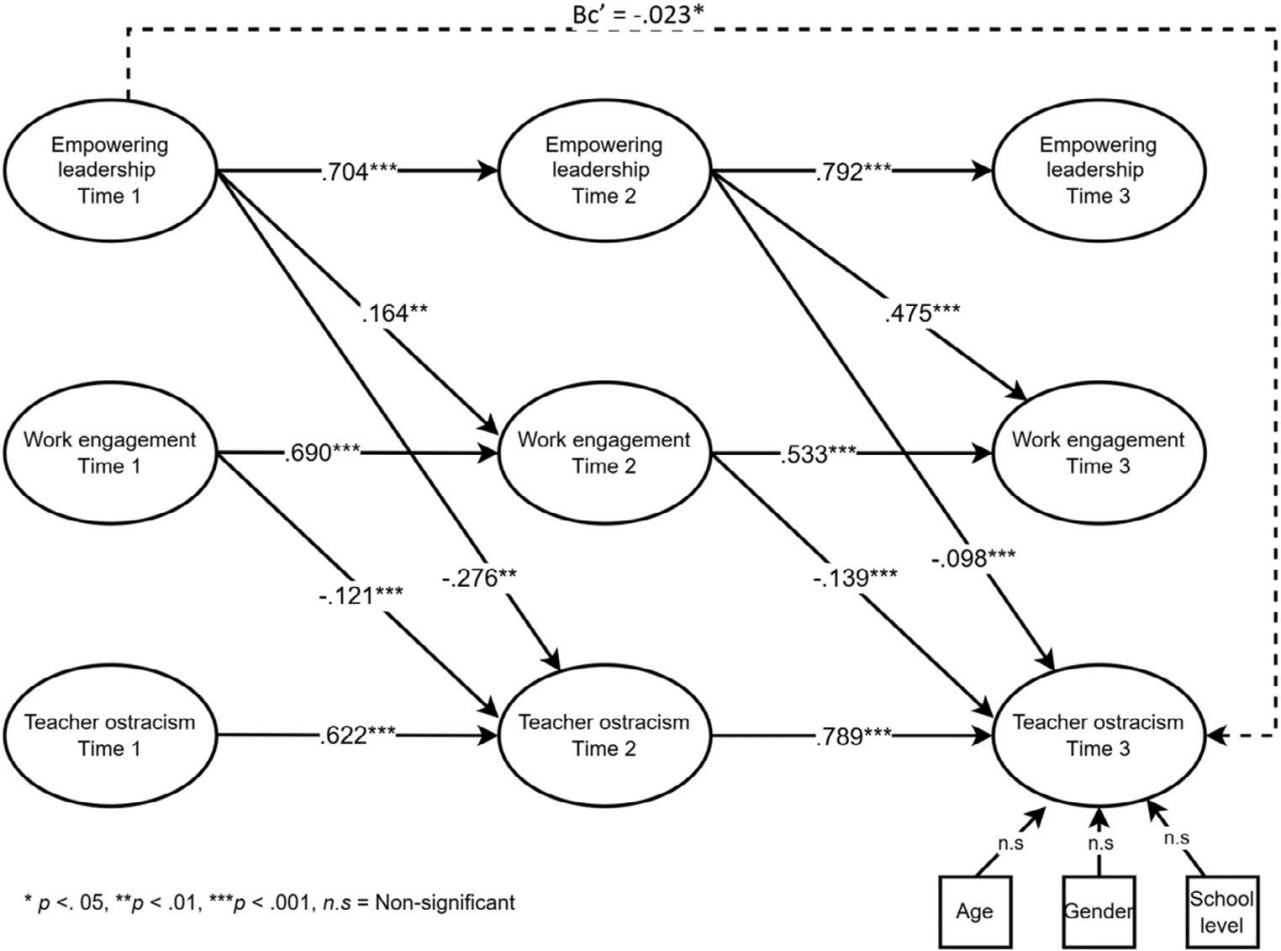
Cross‐lagged panel model estimates.

For model M4^a^, constructed within the framework of the hypotheses and including control variables, standardized parameter estimates obtained from 2000 bootstraps are presented in Figure 2.

The results show that the autoregressive coefficients for empowering leadership (*φ*_T1→T2_ = .704, 95% CI [.618, .780]; *φ*_T2→T3_ = .792, 95% CI [.754, .837]), work engagement (*φ*_T1→T2_ = .690, 95% CI [.564, .777]; *φ*_T2→T3_ = .533, 95% CI [.427, .607]), and teacher ostracism (*φ*_T1→T2_ = .622, 95% CI [.521, .709]; *φ*_T2→T3_ = .789, 95% CI [.427, .607]) were relatively high. The cross‐lagged coefficients revealed that changes in work engagement were positively predicted by residual changes in empowering leadership from Time 1 to Time 2 (*β*_T1→T2_ = .164, 95% CI [.066, .260]) and from Time 2 to Time 3 (*β*_T2→T3_ = .475, 95% CI [.298, .467]), controlling for teacher ostracism, thus confirming Hypothesis 1. Similarly, changes in teacher ostracism were negatively predicted by residual changes in empowering leadership from Time 1 to Time 2 (*β*_T1→T2_ = −.276, 95% CI [−.427, −.148]) and from Time 2 to Time 3 (*β*_T2→T3_ = −.098, 95% CI [−.131, −.068]), controlling for work engagement, confirming Hypothesis 2.

Further, the analysis showed that changes in teacher ostracism were negatively predicted by residual changes in work engagement from Time 1 to Time 2 (*β*_T1→T2_ = −.121, 95% CI [−.207, −.032]) and from Time 2 to Time 3 (*β*_T2→T3_ = −.139, 95% CI [−.185, −.097]), controlling for empowering leadership, confirming Hypothesis 3. In addition, empowering leadership at Time 1 indirectly predicted reductions in teacher ostracism at Time 3 through changes in work engagement at Time 2. The indirect prediction was significant (*β*c′ = −.023, 95% CI [−.058, −.014]), confirming the mediated relationship hypothesized in Hypothesis 4. The effect size calculations indicated that the effect of empowering leadership on work engagement (*r* = .260) and on teacher ostracism (*r* = −.196) corresponded to a medium level of statistical effect. Additionally, the effect of work engagement on teacher ostracism (*r* = −.167) and the effect of empowering leadership on teacher ostracism through work engagement (*r* = −.132) also corresponded to a medium level of statistical effect.

## DISCUSSION

Ostracism, commonly encountered in high‐contact professions like teaching (Williams et al., 2000), poses significant risks to teachers, students, and schools (Uslukaya & Demirtaş, 2020). In collectivist societies like Turkey, ostracism is used as a control mechanism (Scott & Duffy, 2015), with its effects intensified by mutual social dependence (Sato et al., 2014). This has increased academic interest in teacher ostracism and the need for preventive measures. Based on the JD‐R model, this study examines the relationship between empowering leadership and ostracism, both directly and through work engagement, using longitudinal data from 473 Turkish teachers across three time points. The results showed that all hypotheses and the model predicted within the framework of the hypotheses were supported. More specifically, it was determined that empowering leadership is positively related to work engagement and negatively related to teacher ostracism. This suggests that when leaders provide resources such as autonomy, support, and feedback—acting as empowering leaders (Cheong et al., 2019)—teachers can become more engaged in their work and experience less ostracism. These findings highlight the important role that empowering leadership can play in promoting a positive work environment and encouraging positive workplace behaviours while also suggesting its potential to prevent negative workplace experiences. Additionally, the current study predicted that work engagement could be negatively related to teacher ostracism and that there may be a mediating role in the relationship between empowering leadership and teacher ostracism. This result implies, as Bakker and Demerouti (2018) suggest, that engaged employees with high energy and motivation are able to build a positive behavioural cycle in their environments and thus shape the behaviours they will be exposed to. Moreover, it demonstrates that work engagement might serve as a linking mechanism that can further reduce the likelihood of ostracism in environments where empowering leadership is present. The theoretical and practical implications of the current study are discussed below.

### Theoretical contributions

Despite the risks of ostracism in the educational context and the potential theoretical importance of empowering leadership in preventing ostracism in schools, the literature on educational administration has largely overlooked the relationship between empowering leadership and teacher ostracism. This study addresses the calls for research on the leadership‐ostracism relationship (Fiset & Boies, 2018) and the calls in the JD‐R literature to investigate the impact of empowering leadership on employee well‐being and workplace behaviours (Bakker & Demerouti, 2017) by providing evidence from the educational context about the relationship between empowering leadership and ostracism. Thus, it fills a gap in both the educational management and general management literature.

Additionally, this study advances the traditional perspective on ostracism (Howard et al., 2020), which views it as a static condition and primarily focuses on ways to manage its effects, by demonstrating that ostracism is a preventable condition. The findings of the current study are consistent with the previous general literature emphasizing the importance of contextual relationships in reducing employees' feelings of ostracism and being ignored (Robinson et al., 2013; Scott & Duffy, 2015). Moreover, it supports the findings of the few past studies that have cross‐sectionally demonstrated the negative relationship between positive leadership (ethical leadership and transformational leadership) and workplace ostracism (Fiset & Boies, 2018; Kanwal et al., 2019).

Previous studies that theorize the relationship between leadership and ostracism often rely on social learning and social exchange theories (Babalola et al., 2017; Kanwal et al., 2019). This study, however, approaches the leadership‐ostracism relationship from a workplace context by utilizing the JD‐R model. In doing so, it provides evidence for the applicability of integrating ostracism into the JD‐R model and offers a detailed theorization of the negative relationship between leadership and ostracism. Given that the JD‐R model is commonly used to investigate employee well‐being and behaviours (Bakker et al., 2023), this study may encourage future research to utilize the JD‐R model to explore ostracism further.

Consistent with the assumptions of the JD‐R model, the results show a negative longitudinal relationship between empowering leadership and teacher ostracism. This finding supports the view that empowering leadership is generally associated with positive outcomes (Lee et al., 2018), but it contradicts previous studies suggesting that giving employees too much authority and responsibility can be overwhelming for some and lead to negative work outcomes (Cheong et al., 2016). This contradiction can be explained by the cross‐sectional nature of previous studies, as cross‐sectional research captures only the simultaneous relationships between variables at a single point in time (Rindfleisch et al., 2008). Indeed, when done correctly, empowering leadership can function as a job resource, fostering a positive and motivating work environment (Kim et al., 2018) and isolating employees from negative work behaviours by empowering them (Kim & Beehr, 2023). Therefore, this study clarifies doubts about the association between empowering leadership and positive workplace outcomes by demonstrating that it can be linked to isolating employees from negative workplace experiences.

Another theoretical contribution of this study is to theorize and support the relationship between empowering leadership and work engagement. This result supports the JD‐R literature, which predicts that job resources such as leadership will increase employees' work engagement. It also advances the model by integrating empowering leadership into the JD‐R framework. Moreover, although the leadership literature hypothesizes that work engagement is among the potential outcomes of empowering leadership (Spreitzer, 1995), this relationship has generally been supported by cross‐sectional studies (Cai et al., 2018; Wen et al., 2023). Therefore, this study provides robust empirical evidence for this relationship by demonstrating that empowering leadership can predict work engagement over time.

It was concluded that work engagement may be associated with a reduction in ostracism over time. In addition to the well‐known association with positive workplace outcomes, it seems that work engagement can protect teachers from ostracism. This supports the JD‐R literature, which suggests that engaged employees, as active contributors, can foster supportive behaviours and cultivate high‐quality work relationships; thereby they may influencing the behaviours they encounter (Bakker & Demerouti, 2018). This also supports the ostracism literature, which argues that passive, submissive, low‐performing individuals who lack personal power are more likely to be ostracized (Aquino & Lamertz, 2004; Howard et al., 2020; Khan et al., 2018).

Additionally, it was found that work engagement may serve as a linking mechanism in the relationship between empowering leadership and ostracism; that is, it may also have a mediating function. This result supports the mediation pathway of the JD‐R model, which predicts that job resources, such as leadership, can be associated with employee behaviour through certain attitudes (i.e., work engagement) (Schaufeli & Taris, 2014). Additionally, it supports the mediation model of empowering leadership, which predicts that the relationship between empowering leadership and job outcomes may not always be linear and can occur through certain mediators (Cheong et al., 2019). Previous research has tested the mediating roles of variables such as self‐efficacy, passion, and collaboration in the relationship between empowering leadership and job outcomes (Cheong et al., 2016; Hao et al., 2018; Hill & Bartol, 2016). However, the role of work engagement has been overlooked. Therefore, this study advances the model by integrating work engagement into the mediation model of empowering leadership and also expands the nomological network of work engagement.

### Practical implications

This study demonstrates that policymakers should recognize the importance of empowering leadership by school principals, as it may enhance positive work attitudes and reduce negative work behaviours in schools. The results reveal that empowering leadership behaviours of school principals could be an effective means of increasing teachers' work engagement and decreasing the ostracism they experience. Therefore, characteristics such as a tendency to share power, displaying supportive attitudes, and valuing teachers' autonomy should be considered when selecting school principals. Additionally, given the potential impact of empowering leadership behaviours on school outcomes and the fact that these behaviours can be developed (Cheong et al., 2019), policymakers could promote these behaviours by organizing seminars or training sessions for school principals (Yukl, 2012). This approach may help increase teachers' work engagement and prevent ostracism in schools.

School principals should also be aware of the relationship between empowering leadership behaviours and positive workplace outcomes in the school environment and act as empowering leaders. For instance, principals should involve teachers in decision‐making processes, avoid imposing restrictions, provide opportunities for professional development, and offer guidance. Additionally, they should support teachers in managing their work demands and build trust‐based relationships. These behaviours can enhance teachers' work motivation and promote work engagement (Hakanen et al., 2006), thereby potentially contributing to a positive work environment, facilitating high performance, and helping to prevent ostracism (Kim & Beehr, 2023).

In this study, relatively moderate statistical effect sizes were found between the variables. This indicates that the prevention of ostracism can be explained not only by empowering leadership but also by other processes. In other words, the prevention of ostracism may not solely depend on specific leadership behaviours; other leadership approaches may also have an impact. Previous empirical studies have shown that ethical leadership, spiritual leadership, and transformational leadership behaviours are negatively associated with ostracism (Ali et al., 2020; Babalola et al., 2017; Kanwal et al., 2019). In this context, it can be suggested that school principals could develop a more effective strategy for preventing ostracism by diversifying their leadership styles and integrating these approaches.

This study demonstrates that the benefits of work engagement may extend beyond what is currently described in the literature. Policymakers and school principals have various means to increase work engagement, which can reflect the benefits of leadership and may prevent negative work behaviours. Although the literature suggests that work engagement can be enhanced through various interventions, the most effective interventions have been identified as job crafting and mindfulness training (Knight et al., 2019). Therefore, allowing teachers to proactively shape their jobs (job crafting) and providing mindfulness training to help them cope with stress, improve focus, and maintain emotional balance can enhance their engagement in their work. Consequently, this can contribute to creating a more positive and productive school environment (Huang et al., 2022; Tao, 2022), thus aiding in the prevention of ostracism.

### Limitations and suggestions for future research

This study has some limitations. First, the sampling method used can be shown. Longitudinal studies, unlike cross‐sectional studies, can reveal robust relationships between variables; however, they often experience selective attrition, which can hinder the accurate estimation of these relationships. For this reason, a convenience sampling method was preferred in this study to prevent selective attrition. This also points to the need to be careful about the generalizability of research results (Gliner et al., 2011). Another notable limitation of this study is the use of teacher self‐report scales. Although some researchers prefer the use of teacher self‐reports when measuring leadership practices (Thoonen et al., 2011), as Van Dierendonck and Dijkstra (2012) note, a dyadic design in which both leaders and followers provide information about the relationship may increase objectivity. Therefore, it is recommended that future studies model the relationship between school leadership and teacher ostracism using data collected from both leaders and followers.

Another limitation is the use of a single‐level analysis in this study. Since the ICC values for all key variables were estimated to be lower than the cut‐off point of .05, multilevel analyses could not be conducted (Hox et al., 2017). However, analysing models with contextual variables at the individual level may miss contextual relationships. Therefore, it is recommended to analyse multilevel models that examine the relationships between key variables in the future. Finally, the main focus of this study was to investigate the mechanisms that contribute to the prevention of ostracism. Therefore, the hypotheses were formulated accordingly. However, Cheong et al. (2019) suggest that the outcomes of empowering leadership can also serve as predictors. Thus, it is strongly recommended to conduct a comprehensive study using a full cross‐lagged panel model in the future to examine the causal and reverse causal relationships between empowering leadership, work engagement, and ostracism.

## CONCLUSIONS

Despite significant evidence of harmful effects on teachers, students, and schools (Uslukaya & Demirtaş, 2020), the dynamics and connection mechanisms that could prevent teacher ostracism have been overlooked. This study, utilizing the conceptual framework of the JD‐R model, attempts to fill the gap in the literature by explaining the role of empowering leadership in preventing teacher ostracism. Analysis of data collected from teachers in three waves revealed that empowering leadership can reduce teacher ostracism both directly and indirectly by increasing the level of engagement. According to this study, empowering leadership not only benefits teachers but also has the potential to prevent negative work experiences in schools. Based on these findings, various recommendations are made to policymakers and school administrators regarding increasing teacher work engagement and reducing the experience of ostracism.

## AUTHOR CONTRIBUTIONS

**Alper Uslukaya:** Conceptualization; investigation; funding acquisition; writing – original draft; methodology; validation; visualization; writing – review and editing; software; formal analysis; project administration; data curation; supervision; resources.

## CONFLICT OF INTEREST STATEMENT

The author declares that they have no conflict of interest.

## ETHICAL APPROVAL

All procedures performed were in accordance with the ethical standards of the institutional research committee and with the 1964 Helsinki Declaration and its later amendments.

## Data Availability

The data that support the findings of this study are available on request from the corresponding author. Supplementing materials can be accessed via https://figshare.com/s/2996e859c34121234cfa.
